# Moving towards openness: introduction to the registered reports and data notes article collection

**DOI:** 10.1080/21642850.2025.2529274

**Published:** 2025-07-14

**Authors:** E. Toomey, E. Norris

**Affiliations:** a Centre for Health Research Methodology, School of Nursing and Midwifery, University of Galway, Galway, Ireland; b Department of Health Sciences, Brunel University, UK

Improving the transparency and openness of health psychology and behavioural medicine research is of critical importance. This research has a profound impact on people’s lives, and the majority of this research is also publicly funded. Therefore, ensuring transparency in health psychology and behavioural medicine research is essential to make the most of limited research resources and to deliver the greatest possible benefits to individuals and society. However, as with many areas of research, issues with scientific quality and integrity such as publication bias and reproducibility issues exist in this field (Aert et al., 2019). As such, recent times have seen increasing interest in the use of open science practices within health psychology and behavioural medicine (Kwasnicka et al., 2021; Segerstrom et al., 2023).

Achieving open science practices requires multi-faceted interventions targeting multiple levels from individual researchers to research institutions (Silverstein et al., 2024; Zečević et al., 2021). Journals are also an important stakeholder with a valuable role in facilitating open science practices by researchers. However, a recent audit of health psychology and behavioural medicine journal policies showed that the adoption of Transparency and Openness Promotion (TOP) standards (Nosek et al., 2015) such as the incorporation of reporting guidelines, study pre-registration and data transparency requirements within journal policies have been poorly adopted to-date (Toomey et al., 2025).

Registered Reports and Data Notes are two initiatives that journals can consider introducing to improve open science practices. Registered Reports are a type of empirical article conducted in two stages, in which the study’s methods and planned analyses are peer-reviewed and approved (Stage 1) before the research is carried out (Stage 2). Acceptance of the final submission is guaranteed provided the pre-registered methods and analytic procedures are followed. Data Notes are brief, peer-reviewed articles that describe a research dataset made available in an open repository. These article formats can play an important role in improving research transparency and data accessibility, by minimising publication bias and enhancing reproducibility. In May 2024 we launched a call for Registered Reports and Data Notes as part of a special Article Collection within this journal, Health Psychology and Behavioural Medicine (HPBM) (Norris et al., 2024). The purpose of this Article Collection was to highlight the introduction of these new article formats by the journal, and to attract, showcase and house examples of both formats. This editorial provides a brief overview of the current content of the collection, and to emphasise the importance and implications of this collection in advancing research practices within this field.

At the time of writing, the collection consists of six published articles. In addition to launching and promoting the call widely, over 100 researchers in the field were invited to submit to the collection. In total, ten submissions were received; one of these was rejected, one was withdrawn, one is an accepted Stage 1 Registered Report, and one is a protocol for the development of reporting guidelines for qualitative Data Notes which is currently still under review (Long, Toomey, et al., 2025). Of the six published articles, one of these is a methods paper and five are Data Notes. The methods paper was written by the Article Collection’s guest advisors (Toomey and Norris) with colleagues from the European Health Psychology Society (EHPS) Open Science Special Interest Group as part of the initial call for papers (Norris et al., 2024). This article describes what Registered Reports and Data Notes are and the advantages and challenges of each. The piece also provides specific practical guidance on factors to consider for both authors and reviewers in preparing, submitting and/or reviewing these types of articles. Taken with the articles in this collection to serve as examples, this paper aims to facilitate the submission of further such article types within HPBM and elsewhere.

The five Data Note articles featured in the collection describe a variety of datasets of differing sizes and methodological approaches spanning a wide range of clinical topics. For example, Savani et al. describe a large two-wave dataset of over 42,000 survey responses on the behavioural drivers of COVID-19 vaccinations, taken from nationally representative samples of all G-7 countries (Savani et al., 2025). This Data Note provides in-depth description of this sample as well as the methods and measures used to collect the data. The authors also highlight examples of future research that could be conducted with the data. König et al. describe a dataset containing survey data from 273 individuals living in rural Germany which examines the barriers and facilitators to healthy eating and physical activity within rural populations, as well as their engagement with digital health interventions (König et al., 2025). The authors provide detailed information on the dataset population and the measurement tools used, as well as highlighting specific opportunities for further use of the dataset by other researchers. Segerstrom and Kasarskis provide a description of the Seattle Amyotrophic Lateral Sclerosis (ALS) Patient Project Database (SALSPPD), a longitudinal dataset of 143 people with ALS and their partners, with data collection starting in 1987 and continuing until 2008 (Segerstrom & Kasarskis, 2024). The dataset serves as a valuable resource for researchers interested in the psychosocial aspects of chronic illness, caregiving dynamics, and the progression of ALS, particularly given its longitudinal and dyadic nature. The authors specifically comment that while several publications have emerged from the dataset, these *‘only scratch the surface of the dataset’,* and as such provide rich grounds for further research studies. Lloyd and Smith present a dataset derived from interviews with 15 individuals diagnosed with Lynch syndrome and 23 healthcare professionals (HCPs) on the use of aspirin for cancer prevention in this population (Lloyd & Smith, 2025). Of these, fully anonymised interview transcripts from 12 people with Lynch syndrome and eight general practitioners were deposited in a restricted access repository. As well as describing the study population and the data collection methods in-depth, the authors also discuss the limitations of their dataset. Specifically, they acknowledge that by ensuring data were fully anonymised for the dataset, this may have compromised the richness of data deposited and its potential future reuse. As such, this article not only provides insight on how to reuse a valuable resource exploring aspirin use for cancer prevention from both patient and HCP perspectives, but also on the methodological challenges surrounding qualitative data sharing. Finally, Long et al. describe another dataset derived from a qualitative study focusing on HCPs’ experiences in managing cases of false positive breast cancer screening results in women (Long, Branney, et al., 2025). In contrast to Lloyd and Smith (Lloyd & Smith, 2025), this Data Note focuses mostly on data analysis methods and the processed data (e.g. coding and theme development), rather than raw interview transcripts. In doing so, they provide a comprehensive worked example of the template analysis method of qualitative analysis. However, like Lloyd and Smith (Lloyd & Smith, 2025), Long et al. also discuss the challenges of balancing qualitative data sharing with participant privacy (Long, Branney, et al., 2025).

By providing clear descriptions of these varied datasets, the Data Note articles in the Article Collection highlight the value of Data Notes for facilitating transparent and collaborative research that maximises the time and effort that both researchers and research participants have put into generating the datasets in the first place. Although data sharing is extremely valuable and increasingly required by funders and others (Horizon Europe 2021, OSTP Issues Guidance to Make Federally Funded Research Freely Available Without Delay), it is still not routine or expected practice (Hussey, 2023). All too often we see or experience limited mention of data availability, or ‘data available on reasonable request’, with subsequent reasonable requests to authors often ignored (Hussey, 2023). Rather than ‘guarding’ their datasets or research ideas for their own use, the authors of these Data Notes highlight future research projects that other researchers can draw on and use pre-existing data. This represents the altruistic mindset which is needed to transition to a culture of more open, collaborative, efficient and impactful research.

Of note, two of the Data Note articles pertain to qualitative datasets (Lloyd & Smith, 2025; Segerstrom & Kasarskis, 2024), and three to quantitative datasets (König et al., 2025; Long, Branney, et al., 2025; Savani et al., 2025). Both qualitative Data Notes provide exemplars of the use of open science practices within qualitative research. While acceptance of the importance of quantitative data sharing is growing, qualitative data sharing remains challenging and sometimes controversial (Prosser et al., 2024), and data sharing guidance, examples and requirements often pertain to quantitative data only (Branney et al., 2023). This is typically due to very legitimate concerns regarding participant privacy (Campbell et al., 2023); however, the two qualitative Data Note articles in this collection both highlight the need to consider participant privacy alongside other factors such as maximising participant input and minimising over-researching, particularly in smaller or rare disease populations. The contribution made by Segerstrom and Kasarskis’s Data Note describing data collected from the ALS population further reinforces this crucial point (Segerstrom & Kasarskis, 2024). As well as facilitating data sharing, the Data Note articles in the Article Collection also facilitate greater transparency of research methods and the reuse of existing measures and tools. The methods sections in published papers are typically concise summaries which don’t provide sufficient scope to fully elucidate the data collection and analysis approaches. This limits our ability to understand, learn from and replicate those methodologies, as well as hindering data extraction and synthesis in evidence syntheses. The Data Notes in this collection provide much-needed additional information to supplement full scientific articles.

Despite the success of the Article Collection as a showcase for Data Notes however, the proverbial elephant in the open science room must be acknowledged. This is, of course, that none of the current articles are Registered Reports. Despite active promotion and explicit calls targeting both article formats, at the time of writing only one Stage 1 Registered Report has been submitted and accepted within this collection (Coyne et al., 2025); however, this article will not be published until the Stage 2 article is submitted and accepted. This aligns with Taylor & Francis publisher policy where both Stage 1 and Stage 2 articles must be integrated to avoid duplication of content. However, other journals do publish Stage 1 reports (Jach et al., 2025; Jarke et al., 2022), reflecting some of the variability in how Registered Reports are currently implemented across various journals. This lack of a standardised approach for dealing with Registered Reports across journals may be a potential source of confusion for researchers and therefore hinder their uptake. This aligns with views that a lack of awareness and understanding of Registered Reports by researchers is a key barrier to their uptake. It is also crucial that everyone involved in producing a Registered Report fully understands the process, from publishers to editors, authors, reviewers and readers. it is notable that some challenges were also experienced by the editorial staff in handling the Stage 1 Registered Report submitted to this Article Collection and managing it within the existing workflow. This likely added additional time to the process, and as outlined by Norris et al. in this Article Collection (Norris et al., 2024), a barrier to the uptake of Registered Reports by researchers is the perceived time required, particularly for projects and researchers or students with short timelines. As such, to facilitate the uptake of more Registered Reports, training, education and awareness-raising initiatives should target all relevant stakeholders. This should also include research institutions whose promotion and hiring criteria play a key role in incentivising and encouraging open science practices, to ensure such article formats are acknowledged as important academic outputs alongside other metrics used to evaluate academic performance.

As such, this Article Collection plays a vital role in promoting open science and methodological rigour by showcasing several examples of Data Note articles. It also provides a dedicated home for Registered Reports in the field of health psychology and behavioural medicine of which we hope to see more of in coming months. In addition, it provides practical guidance on what these formats are and how to adopt them from both author and reviewer perspectives. We would encourage readers to engage with the collection, consider adopting similar practices in their work and submitting their Data Notes or Registered Reports to the collection. We would like to see this collection expand in time and become a comprehensive repository of high-quality examples of both article formats for this field. We also aim for this collection to provide valuable insights for other journals, both in the area of health psychology and behavioural medicine and beyond. We encourage all journals, institutions, and researchers to consider these article formats as important outputs, and valuable initiatives to enhance research integrity.

## Author contributions

Elaine Toomey: Conceptualisation, Writing Original Draft, Writing – Review & Editing. Emma Norris: Conceptualisation, Writing – Review & Editing.

## References

[CIT0001] Aert, R. v., Wicherts, J. M., & Assen, M. v. (2019). Publication bias examined in meta-analyses from psychology and medicine: A meta-meta-analysis. *PLOS ONE*, *14*(4), e0215052. 30978228 10.1371/journal.pone.0215052 PMC6461282

[CIT0002] Branney, P., Brooks, J., Kilby, L., Newman, K., Norris, E., Pownall, M., Talbot, C.V., Treharne, G. J., & Whitaker, C. M. (2023). Three steps to open science for qualitative research in psychology. *Social and Personality Psychology Compass, 17*(4). doi:10.1111//spc3.12728

[CIT0003] Campbell, R., Javorka, M., Engleton, J., Fishwick, K., Gregory, K., & Goodman-Williams, R. (2023). Open-science guidance for qualitative research: An empirically validated approach for de-identifying sensitive narrative data. *Advances in Methods and Practices in Psychological Science*, *6*(4). doi:10.1177/25152459231205832

[CIT0004] Coyne, R., Varol, T., Phillips, A., O’Mahony, A., Reynolds, J., Grant, S. P., Talsania, K., Calvert, S., Toomey, E., & Norris, E. *Awareness and prevalence of Open Science behaviours among health psychology researchers*. OSF; 2025. Awareness and prevalence of Open Science behaviours among health psychology researchers: A Registered Report. Available from: https://osf.io/aq6e7.

[CIT0005] Horizon Europe. (2021). *Horizon Europe, open science: Early knowledge and data sharing, and open collaboration [Internet]*. Publications Office of the European Union. [cited 2025 May 29]. Available from: https://data.europa.eu/doi/10.277718252.

[CIT0006] Hussey, I. (2023). Data is not available upon request [Internet]. OSF; 2023 [cited 2025 May 29]. Available from: https://osf.io/jbu9r_v1.

[CIT0007] Jach, H. K., Damian, R. I., & Murayama, K. (2025). “The more I learn, the more I know nothing”: A longitudinal registered report of doctoral students’ uncertainty in learning. *Learning and Instruction*, *98*, 102138. doi:10.1016/j.learninstruc.2025.102138

[CIT0008] Jarke, H., Jakob, L., Bojanić, L., Garcia-Garzon, E., Mareva, S., Mutak, A., & Gjorgjiovska, J. (2022). Registered report: How open do you want your science? An international investigation into knowledge and attitudes of psychology students. *PLOS ONE*, *17*(2), e0261260. doi:10.1371/journal.pone.0261260 35226677 PMC8884512

[CIT0009] König, L. M., Betz, C., Masri, M. A., & Bartelmeß, T. (2025). Health behaviours and mobile intervention use in patients recruited from general practitioners’ practices in rural Bavaria. *Health Psychology and Behavioral Medicine*, *13*(1). https://www.tandfonline.com/doi/abs/10.108021642850.2024.2444244. 10.1080/21642850.2024.2444244 PMC11703449 39777054

[CIT0010] Kwasnicka, D., ten Hoor, G. A., van Dongen, A., Gruszczyńska, E., Hagger, M. S., Hamilton, K., Hankonen, N., Heino, M. T. J., Kotzur, M., Noone, C., Rothman, A. J., Toomey, E., Warner, L. M., Kok, G., Peters, G. -J., & Luszczynska, A. (2021). Promoting scientific integrity through open science in health psychology: Results of the Synergy Expert Meeting of the European health psychology society. *Health Psychology Review*, *15*(3), 333–49. doi:10.1080/17437199.2020.1844037 33198583

[CIT0011] Lloyd, K. E., & Smith, S. G. (2025). Dataset for a qualitative interview study exploring the barriers and facilitators to using and recommending aspirin for cancer prevention. *Health Psychology and Behavioral Medicine*, *13*(1), 2463916. doi:10.1080/21642850.2025.2463916 39959432 PMC11827028

[CIT0012] Long, H. A., Branney, P., French, D. P., & Brooks, J. M. (2025). Optimising data sharing whilst protecting participant privacy: A data note describing processed data from a qualitative study of healthcare professionals’ experiences of caring for women with false positive screening test results. *Health Psychology and Behavioral Medicine*, *13*(1), 2449400. doi:10.1080/21642850.2024.2449400 39807207 PMC11727048

[CIT0013] Long, H. A., Toomey, E., Stevenson, F., Brooks, J. M., Stewart, A. J., & French, D. P. (2025). Developing a Data Note reporting guideline for qualitative health and social care research datasets (the DeNOTE study): A study protocol [Internet]. medRxiv; 2025 [cited 2025 May 29]. p. 2025.02.04.25321640. Available from: https://www.medrxiv.org/content/10.11012025.02.04.25321640v1. 10.1080/21642850.2025.2532792 PMC12281645 40697405

[CIT0014] Norris, E., O’Mahony, A., Coyne, R., Varol, T., Green, J. A., Reynolds, J., & Toomey, E. (2024). Demystifying Open Science in health psychology and behavioral medicine: A practical guide to Registered Reports and Data Notes. *Health Psychology and Behavioral Medicine*, *12*(1). https://www.tandfonline.com/doi/abs/10.108021642850.2024.2351939. 10.1080/21642850.2024.2351939 PMC11138224 38817594

[CIT0015] Nosek, B. A., Alter, G., Banks, G. C., Borsboom, D., Bowman, S. D., Breckler, S. J., Buck, S., Chambers, C. D., Chin, G., Christensen, G., Contestabile, M., Dafoe, A., Eich, E., Freese, J., Glennerster, R., Goroff, D., Green, D. P., Hesse, B., Humphreys, M., ... Yarkoni, T. (2015). Promoting an open research culture. *Science*, *348*(6242), 1422–5. doi:10.1126/science.aab2374 26113702 PMC4550299

[CIT0016] OSTP Issues Guidance to Make Federally Funded Research Freely Available Without Delay. The White House. 2022 [cited 2025 May 29]. Available from: https://bidenwhitehouse.archives.gov/ostp/news-updates/2022/08/25/ostp-issues-guidance-to-make-federally-funded-research-freely-available-without-delay/.

[CIT0017] Prosser, A. M., Bagnall, R., & Higson-Sweeney, N. (2024). Reflection over compliance: Critiquing mandatory data sharing policies for qualitative research. *Journal of Health Psychology*, *29*(7), 653–8. doi:10.1177/13591053231225903 38282356 PMC11141091

[CIT0018] Savani, M. M., Banerjee, S., Hunter, A., John, P., Koenig, R., Lee-Whiting, B., Loewen, P., McAndrews, J., & Nyhan, B. (2025). What nudges you to take a vaccine? Understanding behavioural drivers of COVID-19 vaccinations using large-scale experiments in the G-7 countries. *Health Psychology and Behavioral Medicine [Internet]*, *13*(1). https://www.tandfonline.com/doi/abs/10.108021642850.2025.2490550. 10.1080/21642850.2025.2490550 PMC12004716 40248162

[CIT0019] Segerstrom, S. C., Diefenbach, M. A., Hamilton, K., O’Connor, D. B., & Tomiyama, A. J. (2023). Behavioral Medicine Research Council. Open science in health psychology and behavioral medicine: A statement from the behavioral medicine research council. *Annals of Behavioral Medicine*, *57*(5), 357–67. doi:10.1093/abm/kaac044 37010262

[CIT0020] Segerstrom, S. C., & Kasarskis, E. J. (2024). The Seattle Amyotrophic Lateral Sclerosis (ALS) patient project database: Observational, longitudinal, dyadic characterization of people with ALS and their partners. *Health Psychology and Behavioral Medicine*, *12*(1), 2396137. doi:10.1080/21642850.2024.2396137 39239358 PMC11376292

[CIT0021] Silverstein, P., Pennington, C. R., Branney, P., O’Connor, D. B., Lawlor, E., O’Brien, E., & Lynott, D3. (2024). A registered report survey of open research practices in psychology departments in the UK and Ireland. *British Journal of Psychology*, *115*(3), 497–534. https://onlinelibrary.wiley.com/doi/abs/10.1111bjop.12700. 38520079 10.1111/bjop.12700

[CIT0022] Toomey, E., Coyne, R., Derksen, C., Grant, S., Jones, C., Kijowska, M., McNeil, I., Naughton, F., O'Mahony, A., & Norris, E. (2025). Adoption of the Transparency and Openness Promotion (TOP) guidelines within health psychology and behavioural medicine journal policies: a cross-sectional study. *Health Psychology Review*. doi:10.1080/17437199.2025.2516010 40513012

[CIT0023] Zečević, K., Houghton, C., Noone, C., Lee, H., Matvienko-Sikar, K., & Toomey, E. (2021). Exploring factors that influence the practice of Open Science by early career health researchers: a mixed methods study. *HRB Open Research*, *18*(3). https://hrbopenresearch.org/articles/3-56. 10.12688/hrbopenres.13119.1 PMC7836032 33537552

